# Delayed onset of neurosarcoidosis after concurrent ipilimumab/nivolumab therapy

**DOI:** 10.1186/s40425-018-0390-2

**Published:** 2018-07-31

**Authors:** Irena Tan, Michael Malinzak, April K. S. Salama

**Affiliations:** 1 Department of Internal Medicine, Duke University Medical Center, 2301 Erwin Rd, Durham, NC 27701 USA; 2 Department of Neuroradiology, Duke University Medical Center, 2301 Erwin Rd, Durham, NC 27701 USA; 3 Division of Medical Oncology, Duke University Medical Center, 2301 Erwin Rd, Durham, NC 27701 USA

**Keywords:** Ipilimumab, Nivolumab, Immune-related adverse events, Neurosarcoidosis

## Abstract

**Background:**

Immune checkpoint inhibitors have transformed the treatment landscape for many cancers, including metastatic melanoma, but have also opened the door for a diverse variety of immune-related adverse effects.

**Case presentation:**

We describe the first reported case of presumed neurosarcoidosis as an immune-related adverse effect that developed nearly a year after discontinuation of treatment with combination ipilimumab and nivolumab for recurrent metastatic melanoma. The patient was noted to develop clinical signs consistent with systemic sarcoidosis shortly after the initiation of treatment and underwent a biopsy of hilar lymphadenopathy that confirmed sarcoidosis and after which immunotherapy was discontinued. His melanoma remained stable on surveillance imaging for the next year after which time he developed neurological symptoms and was found to have MRI brain abnormalities without evidence of intracranial metastatic disease, consistent with probable neurosarcoidosis given biopsy-proven systemic sarcoidosis and lack of evidence of CNS infection or malignancy. He underwent treatment with high dose steroids, followed by infliximab, and then methotrexate with both clinical and radiographic improvement within 4 months of starting treatment.

**Conclusions:**

Immune-related adverse effects often occur within 3–6 months of receiving immune checkpoint inhibitor therapy, with some reports of late toxicity. This report highlights a case of probable neurosarcoidosis nearly a year after discontinuation of immune checkpoint therapy. The potential for durable responses after discontinuation of therapy also likely underscores a potential for late toxicity. In patients presenting with new or unexplained symptoms after checkpoint inhibitor therapy, the index of suspicion for an immune-related adverse effect should remain high, irrespective of timing.

## Background

The development of novel checkpoint inhibitors, including ipilimumab, a monoclonal antibody against cytotoxic T-lymphocyte-associated antigen-4 (CTLA-4), and the anti-programmed-death 1 (anti-PD1) antibodies nivolumab and pembrolizumab, have transformed the treatment landscape for patients with advanced melanoma [1].

More recently, combination checkpoint blockade has demonstrated considerable promise: responses are seen in a majority of patients, and recently updated analyses suggest these are durable [2]. The unique method with which these therapies upregulate the immune system to cancer cells has also opened the door to a novel class of adverse effects, known as immune-related adverse effects (IRAE). While the most common IRAEs typically manifest themselves early in the course of therapy, and can affect the gastrointestinal, endocrine, and cutaneous systems, serious rare side effects do occur. Sarcoidosis has previously been reported as an adverse effect of checkpoint inhibition [1, 2]. To date, to the authors’ knowledge, there have not been any reports of sarcoidosis as an IRAE on such a delayed timeline as the one seen in this case report [3, 4].

## Case presentation

In 2013, a 65-year-old patient with no prior history of sarcoidosis was diagnosed with a 0.67 mm superficial spreading melanoma on his back. His family history was not significant for autoimmune disease including sarcoidosis and he had a remote 13 pack-year smoking history. He was treated with wide local excision and underwent sentinel lymph node biopsy which was negative. In 2015, he was found to have recurrence of his melanoma with an intensely FDG-avid right axillary lymph node, bilateral pulmonary nodules, and a right adrenal lesion concerning for metastatic disease. There were no abnormalities seen on a brain MRI obtained at that time. Biopsy of the right axillary lymph node confirmed melanoma. He was started on combination ipilimumab 3 mg/kg IV and nivolumab 1 mg/kg IV in October of 2015. After one cycle he developed grade 2 diarrhea which resolved with steroids, however during his steroid taper he developed a grade 2 transaminitis which subsequently resolved with an additional taper. He elected to proceed with the second cycle, and then developed immune-mediated colitis which was refractory to high dose steroids, but resolved after two doses of infliximab 5 mg/kg IV spaced 1 month apart. Shortly thereafter, he developed a rash, arthralgias and hypercalcemia; PET imaging revealed persistent FDG-avid axillary lymphadenopathy, along with new FDG-avid mediastinal and hilar lymphadenopathy. A bronchoscopic biopsy of two mediastinal lymph nodes revealed non-caseating granulomas consistent with sarcoidosis. His symptoms at that time spontaneously resolved without additional treatment. Further immunotherapy was held, and surveillance scans demonstrated stable right axillary adenopathy.

**Fig. 1:**
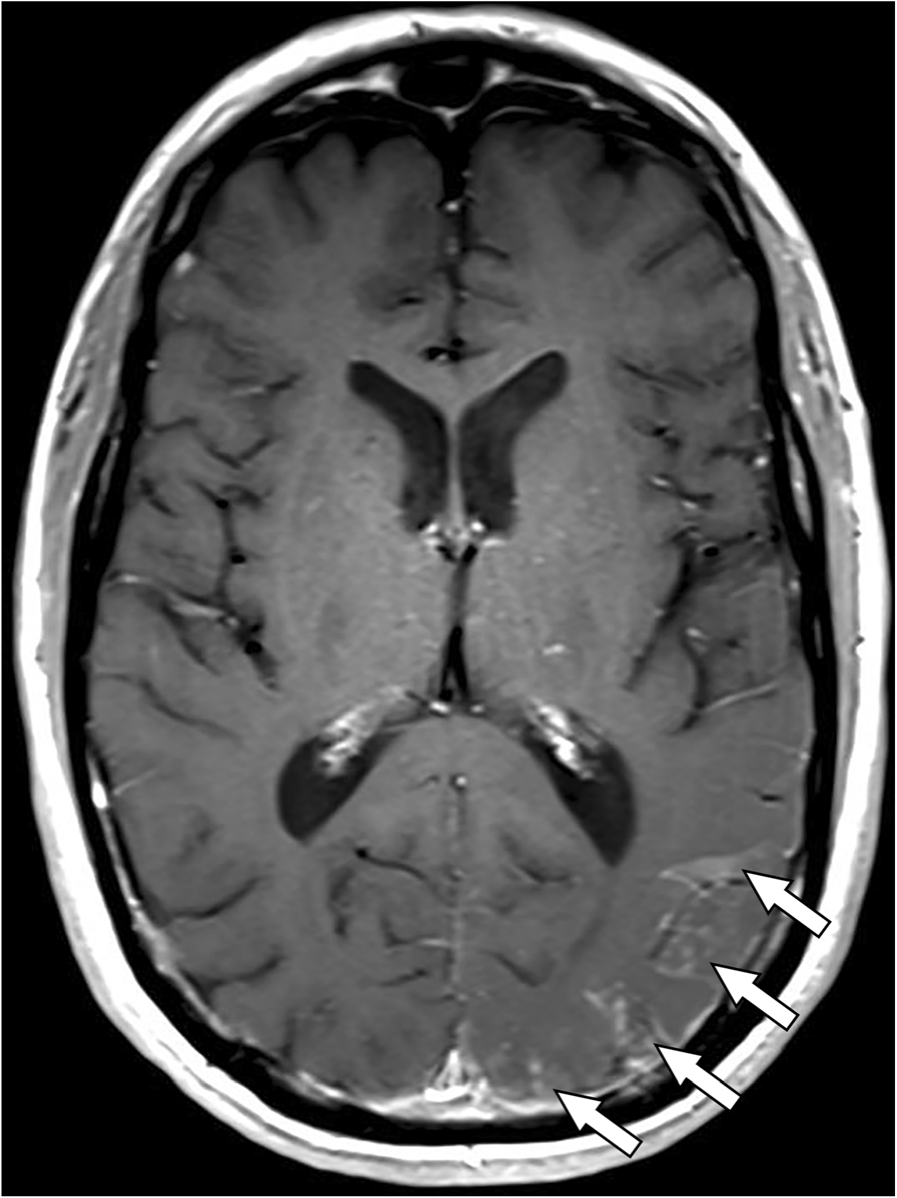
Brain MRI obtained at onset of neurological symptoms. T1-weighted contrast-enhanced axial image shows leptomeningeal enhancement involving the left occipital and parietal lobes (arrows)

**Fig. 2:**
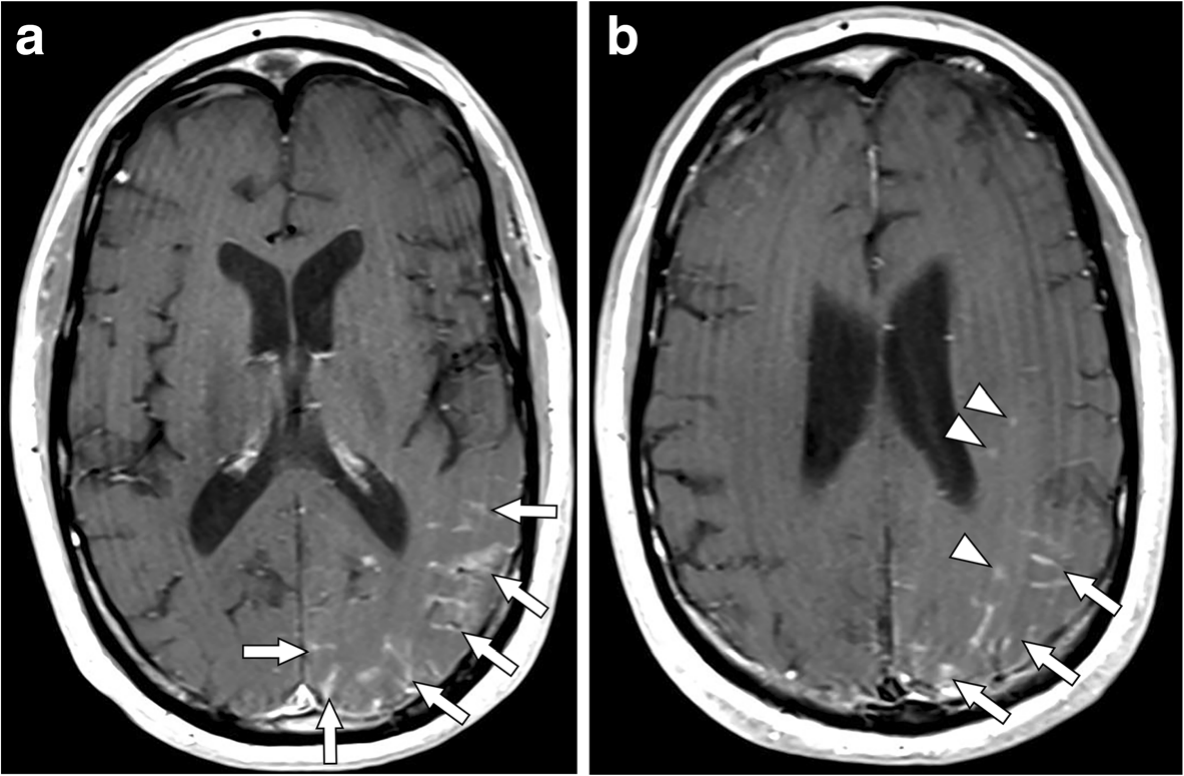
Brain MRI obtained after treatment with steroids in setting of continued neurological complaints. T1-weighted contrast-enhanced axial images at **a** the level of the internal capsule and **b** the level of the corona radiata. Note the increased leptomeningeal enhancement (arrows) and new patchy areas of adjacent parenchymal enhancement (arrowheads)

**Fig. 3:**
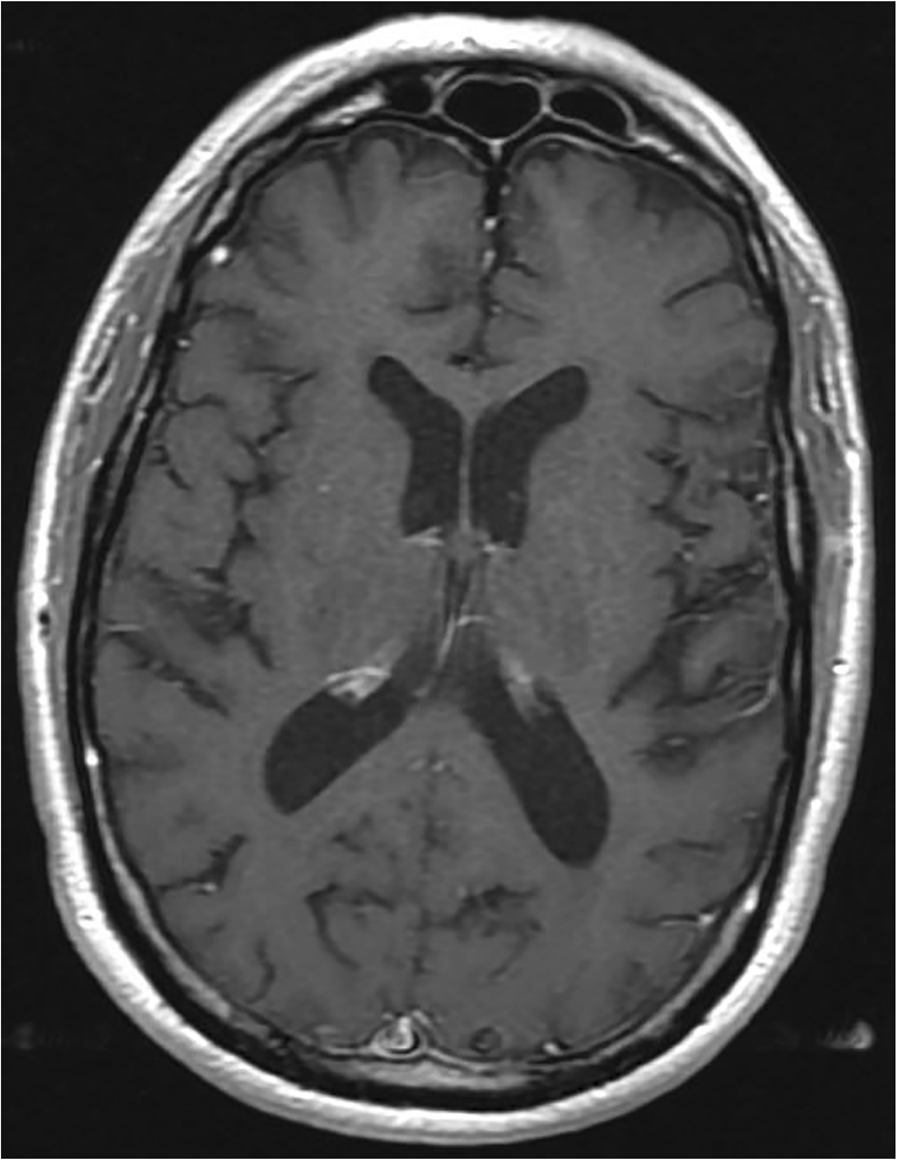
Brain MRI obtained 4 months after starting methotrexate in setting of neurological improvement. T1-weighted contrast-enhanced axial image through the parieto-occipital region shows resolution of leptomeningeal enhancement

However, in October 2016, he presented with transient expressive aphasia lasting less than 30 min. He also noted several weeks of intermittent right-sided visual field deficits. A contrast-enhanced brain MRI demonstrated leptomeningeal enhancement in the left occipital and parietal lobes (Fig. 1), which can be seen with leptomeningeal carcinomatosis, infectious meningitis, or a variety of inflammatory conditions. Spine imaging was not obtained. He then underwent a lumbar puncture which demonstrated elevated protein of 75, normal glucose of 93 (serum glucose 160), a mild pleocytosis with nucleated cell count of 13 (5% neutrophils, 45% lymphocytes), as well as negative cytology studies. No culture studies were sent as the suspicion for infection based on his clinical presentation was low. He was started on high dose dexamethasone 4 mg IV every 6 h due to worsening mental status, which rapidly improved after the start of steroid therapy. Four days after admission, he had a generalized seizure and a repeat cerebrospinal fluid cytology was again negative for melanoma. Combined with the likely pulmonary sarcoidosis seen on his bronchoscopy earlier that year, his neurological symptoms and radiographic studies appeared most consistent with a diagnosis of neurosarcoidosis, likely a rare manifestation of an immune-related adverse effect in the setting of immunotherapy. He was discharged on dexamethasone 4 mg by mouth every 6 h, and given initial improvement in symptoms, his dexamethasone was slowly tapered off over the course of 4 months. In spite of treatment with steroids, he continued to report waxing and waning symptoms of headache, fatigue, and confusion. A repeat contrast-enhanced brain MRI showed increasing leptomeningeal enhancement with new adjacent areas of parenchymal enhancement in a pattern consistent with worsening neurosarcoidosis (Fig. 2). Several weeks after completing his steroid taper, he was initiated on immune modulating therapy with infliximab 5 mg/kg IV accompanied by methylprednisolone 125 mg IV with each infusion, which he received twice every 2 weeks, and then, due to an infusion reaction shortly after beginning his third infusion, transitioned to methotrexate 12.5 mg weekly, which was tapered to 10 mg weekly 3 months after initiation due to mild mood changes. Four months after starting methotrexate, he has had clinical and radiographic improvement (Fig. 3). Additionally, as the right axillary node remained the only site of disease, he underwent an excision; pathology revealed deposits of metastatic melanoma.

## Discussion

Sarcoidosis is an inflammatory disorder in which granulomas develop in a wide array of organ systems, most commonly the lungs, the eyes, and skin [5]. The etiology of this disorder has not been identified, but the pathogenesis of sarcoidosis is thought to stem from an injury or insult, which results in upregulation of granulomatous development [5]. The diagnosis of sarcoidosis is reliant upon the presence of characteristic radiologic and clinical findings, and is further bolstered by histological findings of non-caseating epithelioid-cell granulomas without other obvious external drivers of inflammation (e.g., infection) [5]. In the patient presented in this case report, he had clinical findings (arthralgias, hypercalcemia) and radiologic findings (mediastinal lymphadenopathy) consistent with sarcoidosis, which was supported by non-caseating granulomas seen on histologic examination of a fine-needle aspiration. Histologic evidence of central nervous system (CNS) involvement is seen in up to a quarter of patients known to have systemic sarcoidosis on post-mortem studies, but clinical symptoms are rare [6]. The clinical manifestations of neurosarcoidosis are heterogeneous and symptoms can include visual complaints, meningeal signs, and seizures [6]. The patient described in this report presented with visual field deficits, headache, and was noted to have a first-time seizure. While the initial concern was for the development of metastatic disease, cytology on LP was negative, even after repeat analysis. Sarcoidosis is an uncommon immune-related adverse effect that has been seen with immune checkpoint inhibitor therapy [3, 4]. One case report lists 11 known cases of monotherapy with an immune checkpoint inhibitor leading to known or suspected sarcoidosis as an immune-related adverse event [3], and additionally describes to their knowledge, the first known case of sarcoidosis after combined immunotherapy. Specifically, they observe the development of pulmonary and cutaneous sarcoidosis shortly after a patient with metastatic melanoma completed four cycles of combined ipilimumab and nivolumab therapy [3]. Another case report describes two separate cases of sarcoidosis developing in the wake of receiving anti-PD-1 and anti-CTLA-4 therapy, either simultaneously, or sequentially [4]. Both of these patients developed cutaneous and pulmonary sarcoidosis while actively undergoing treatment with immunotherapy [4]. All of these cases stand in contrast to the patient described in this case report, who continued to show progression of his sarcoidosis through the development of presumed neurosarcoidosis many months after his last dose of immune checkpoint inhibition. In this case report, this patient’s MRI scan demonstrated leptomeningeal enhancement, and at last follow up the patient has clinically improved on immune-modulating therapy, all more consistent with probable neurosarcoidosis. However, it is important to note that granulomas can be seen as a result of malignancy and therefore the granulomas noted on biopsy of his hilar lymphadenopathy may represent either sampling error or sequelae of malignancy, or it is possible that he developed sarcoidosis spontaneously and independent of his treatment with immunotherapy.

While infliximab is considered a therapeutic option for sarcoidosis refractory to steroids, paradoxical reactions can occur with TNF-alpha inhibitors [7]. Case series have reported the development of sarcoidosis after the administration of TNF-alpha inhibitors for other autoimmune disorders. The mechanism by which this occurs is still unknown, but appears related to the disruption of cytokine regulation, particularly an imbalance of cytokines towards interferon and potentially Th17 cytokines [7, 8]. Of note, the patient in our case did receive infliximab therapy twice early on in his course as therapy for a steroid refractory immune mediated colitis. However, it seems more likely that the development of sarcoidosis was a consequence of checkpoint inhibition rather than a reaction to his brief treatment with infliximab, as these paradoxical reactions tend to occur after prolonged courses of biological therapy, and generally resolve after cessation of the TNF-alpha inhibitor [7]. In his case, his symptoms and radiographic findings continued to worsen long after cessation of TNF-alpha inhibition, and actually responded to infliximab therapy.

Whether the development of sarcoidosis in the setting checkpoint inhibition is an unmasking of an underlying autoimmune condition, or a de novo disruption of intrinsically normal immunologic pathways is unknown. There is some evidence that inhibition of CTLA-4 increases Th17 CD4+ cells in the peripheral blood [9], and there is also evidence that tissue samples consistent with sarcoidosis show increased infiltration of Th17 effector CD4+ cells in tissue [10]. In addition, upregulation of PD-1 and PD-L1 have been seen in sarcoid tissue, which would conversely suggest that inhibition of the PD-1 or PD-L1 pathway may actually be beneficial in treatment of sarcoidosis, which is evocative of the paradoxical effects mentioned above with TNF-alpha inhibition [11]. Further investigation on the pathways that overlap between checkpoint inhibition and sarcoidosis will hopefully elucidate the mechanism by which sarcoidosis develops in these patients.

## Conclusions

In general, most IRAE develop within 6 months of receiving their immunotherapy, though the timing is variable. This patient did not develop symptoms concerning for CNS involvement until nearly 11 months after the administration of two courses of ipilimumab-nivolumab therapy, after fully recovering from prior IRAE. In addition, his suspected neurosarcoidosis continued to show progression even several months after diagnosis despite initiation of steroids. This case should raise awareness that serious IRAE may still develop long after the administration of immunotherapy. With expanding indications for immunotherapy in many cancer types, it is more important than ever for patients, oncologists and other care providers to recognize this potential [12]. A greater understanding of the progression of IRAE will become necessary for both their diagnosis and appropriate multi-disciplinary management.

## Abbreviations

CNS: Central nervous system
CTLA-4: Cytotoxic T-lymphocyte antigen 4
FDG: Fludeoxyglucose
IRAE: Immune-related adverse effects
PD-1: Programmed cell death protein 1
PD-L1: Programmed death ligand 1
PET: Positron emission tomography
Th: T helper
TNF-alpha: Tumor necrosis factor alpha
